# Supplementary material for: Body Size and Bite Force of Stray and Feral Cats—Are Bigger or Older Cats Taking the Largest or More Difficult-to-Handle Prey?
Source: Animals (Basel). 2020 Apr 17;10(4):707. doi: 10.3390/ani10040707 (PMC7222765; doi:10.3390/ani10040707)
Supplement: Supplementary file 1 [file animals-10-00707-s001.pdf]

animals

MDPI

Article

Supplementary Files: Body Size and Bite Force of Stray and Feral Cats—Are Bigger or Older Cats Taking the Largest or More Difficult-to-Handle Prey?

Patricia A. Fleming *, Heather M. Crawford, Clare Auckland, and Michael C. Calver

Table S1. Locations where 567 feral and stray cats (*Felis catus*) were sourced across southwest Western Australia between 2010 and 2018.

Rural Locations (Feral cats *n* = 419):	Urban Locations (Stray cats *n* = 148):
Badgingarra	Armadale
Bindoon	Aubin Grove
Boonanaring	Balcatta
Boyagin	Balga
Busselton	Bayswater
Contine	Beaconsfield
Coorow	Bedfordale
Cowaramup	Beldon
Cuballing	Bellevue
Cullalla	Bentley
Darkan	Bibra Lake
Dowerin	Bickley
Dowerin	Bicton
Dryandra	Carabooda
Esperance	Coogee
Gillingarra	Coolbellup
Gingin	Cottesloe
Harvey	Dianella
Kellerberrin	East Victoria Park
Lancelin	Golden Bay
Lennard Brook	Gosnells
Marchagee	Gwelup
Mogumber	Hamersley
Mooliabeenee	Hamilton Hill
Moondah	Hazelmere
Mount Barker	High Wycombe
Mount Caroline	Highgate
Narrikup	Innaloo
Narrogin	Jandakot
Orange Springs	Kardinya
Popanyinning	Martin
Red Gully	Maylands
	Medina
	Munster
	Murdoch
	North Coogee
	O'Connor

**Rural Locations (Feral cats *n* = 419):**	**Urban Locations (Stray cats *n* = 148):**
Seabird<br>Takalarup<br>Torbay<br>Williams	Osborne Park<br>Perth Airport<br>Port Kennedy<br>Sorrento<br>South Guildford<br>Spearwood<br>St James<br>Welshpool

© 2020 by the authors. Licensee MDPI, Basel, Switzerland. This article is an open access article distributed under the terms and conditions of the Creative Commons Attribution (CC BY) license (http://creativecommons.org/licenses/by/4.0/).
